# Zebrafish Astroglial Morphology in the Olfactory Bulb Is Altered With Repetitive Peripheral Damage

**DOI:** 10.3389/fnana.2020.00004

**Published:** 2020-02-11

**Authors:** Jackson Scheib, Christine Byrd-Jacobs

**Affiliations:** Department of Biological Sciences, Western Michigan University, Kalamazoo, MI, United States

**Keywords:** astroglia, astrogliosis, zebrafish, olfactory bulb, GFAP, deafferentation

## Abstract

Zebrafish do not possess the typical astrocytes that are found in mammalian systems. In some brain areas, this teleost has radial glia that appears to perform astrocyte-like functions, but these cells have not been described in the zebrafish olfactory bulb. Mammalian astrocytes facilitate neuroplasticity and undergo astrogliosis after insult. The role of these cells in the zebrafish olfactory system after the damage has been poorly explored. This is important to examine because zebrafish have a high degree of neuroplasticity and the olfactory bulb is a brain area renowned for plasticity. The goal of this study was to explore the potential role of zebrafish astrocytes in the olfactory bulb damage response, with a goal to exploit the high level of regeneration in this system. We found that anti-glial fibrillary acidic protein (GFAP) labels numerous processes in the zebrafish olfactory bulb that are concentrated in the nerve and glomerular layers (GL) and do not show radial glial-like morphology. We propose to term this astroglia, since their location and response to damage suggests that they are similar in function to the mammalian astrocyte. To induce repetitive peripheral damage to the olfactory organ, a wax plug was inserted into the nasal cavity of adult zebrafish every 12 h for up to 7 days; this crushes the olfactory organ and leads to degradation of olfactory sensory neuron axons that project to the olfactory bulb. After 1 day, we found a significant increase in astroglial labeling in the affected bulb when compared to the internal control bulb and astroglial branches appeared to increase in number and size. By the third day of plug insertions there was no significant difference in astroglial labeling between the affected bulb and the internal control bulb. These data lead us to believe that astrogliosis does occur in the presence of peripheral damage, but this process attenuates within 1 week and no glial scar is evident upon recovery from the damage. Further exploration of astrocytes in zebrafish, in particular this apparent attenuation of astrogliosis, has the potential to elucidate key differences in glial function between teleosts and mammals.

## Introduction

Astrocytes are crucial cells in the central nervous system (CNS) that provide energy substrates to neurons and influence synaptic transmission and plasticity (Barros and Deitmer, 2010; Pérez-Alvarez and Araque, 2013; Baldwin and Eroglu, 2017). Since they play a role in neuroplasticity, which is necessary for recovery from injury to the CNS, their activity in the presence of damage is crucial to understand. Insults to the CNS typically cause astrogliosis, which paradoxically can be both neuroprotective and neurotoxic in mammals and, if repetitive, may lead to the development of disease states (Sofroniew, 2009; Pekny et al., 2014; Burda et al., 2016; Kulbe and Hall, 2017; Sullan et al., 2018). Glial scarring may also occur during astrogliosis and is typically thought of as detrimental and neurotoxic, though some components of the glial scar may be beneficial, as it has been shown that it may be needed for axon regeneration (Anderson et al., 2016). Clearly, it would be beneficial to alter some features of astrogliosis and the subsequent scar formation to eliminate neurotoxic aspects and obtain maximum neuroplasticity to recover from injury. One way to approach this problem is to elucidate the cellular and molecular mechanism of neuroplasticity in regenerative model organisms, such as zebrafish, to find key differences in their physiology to exploit for medical intervention in mammals.

Adult zebrafish are renowned for their neuroplasticity and have been shown to recover and regenerate many nervous system structures after damage (Becker et al., 1997; Zupanc, 2008; März et al., 2011). While most cellular aspects of the CNS are comparable to mammalian systems, zebrafish lack permanent glial scaring, like other teleosts (Baumgart et al., 2012; Takeda et al., 2015; Vitalo et al., 2016). This suggests that astrogliosis in zebrafish differs from that in mammals, and this may influence their dynamic neuroplasticity and promote their ability to recover from nervous system insults. Thus, studying astrocytes and astrogliosis in zebrafish has the potential to identify novel physiological processes involved in CNS recovery from damage.

In particular, the adult zebrafish olfactory system is an excellent model to study neuroplasticity due to constant turnover of olfactory sensory neurons (Byrd and Brunjes, 2001; Oehlmann et al., 2004) and the presence of stem cell niches in the olfactory organ and olfactory bulb (Byrd and Brunjes, 2001; Zupanc et al., 2005; Adolf et al., 2006; Grandel et al., 2006). Since synapses are constantly being formed and degraded between olfactory sensory neurons and olfactory bulb neurons, astrocytes within the olfactory bulb likely mediate this process, although zebrafish astrocytes in the olfactory bulb are relatively unexplored, even though similar glial cells have been explored in other brain regions of this fish (Grupp et al., 2010).

Immunohistochemical labeling using antibodies against the glial fibrillary acidic protein (GFAP), a common astrocyte and radial glia marker, shows long, organized processes in the telencephalon of adult zebrafish (Grupp et al., 2010). This labeling pattern is typical of radial glia and, given the lack of stellate-shaped labeling patterns, led many researchers to conclude that zebrafish lack astrocytes and retain radial glia that performs many of the tasks usually reserved for astrocytes. However, in the olfactory bulb of adult zebrafish, GFAP is expressed in processes that seem shorter and less organized than in the telencephalon and do not show the morphology of radial glial or traditional stellate astrocytes (Byrd and Brunjes, 1995). Given that descriptions of these cell types are vague, we propose a definition for the anti-GFAP-immunoreactive cells that fill the astrocyte role in zebrafish. In this study the term “astroglia” will refer to the cells in adult zebrafish that fill the astrocyte role and are likely the most similar in function to a mammalian astrocyte, inferred by their immunohistochemical labeling profile and morphology.

Glutamine synthetase (GS), an enzyme involved in glutamate metabolism, has been shown to be expressed by astrocytes (Norenberg and Martinez-Hernandez, 1979) and has been characterized in the telencephalon of zebrafish (Grupp et al., 2010) but not the olfactory bulb. We speculated that GS will be localized in astroglia in the olfactory bulb and using anti-GFAP and anti-GS antibodies may elucidate their morphology. Therefore, our study first examined astroglia in the olfactory bulb of adult zebrafish based on immunohistochemical reactivity and morphology.

Zebrafish have been shown to lack glial scarring after single, direct lesions to the telencephalon (März et al., 2011; Baumgart et al., 2012), but astroglial reactivity to repetitive injury of a peripheral component has not yet been explored. Furthermore, it is unknown if this damage persists, if astrogliosis will be chronic, and if a glial scar will form. Therefore, once we have identified astroglia in the olfactory bulb, the second aim of this study was to investigate astrogliosis in the olfactory bulb after a repetitive injury to the olfactory organ. We used a method previously characterized by our lab (Scheib et al., 2019) in which a wax plug is inserted into the nasal cavity to crush the olfactory organ every 12 h for 7 days. Briefly, this form of insult results in a loss of olfactory sensory neuron axons in the olfactory bulb, which may be detected by astroglia in the glomeruli.

Our hypothesis was that repetitive peripheral damage from repeated wax plug insertions causes astrogliosis in the olfactory bulb. We examined qualitatively and quantitatively the size and amount of astroglial branches to detect astrogliosis, and we investigated astroglial proliferation. Since previous studies of zebrafish astrogliosis have shown a lack of glial scarring, we also hypothesized that there will be no glial scar if the olfactory system is allowed to recover fully.

## Materials and Methods

### Animals

Adult male and female zebrafish, *Danio rerio*, over 6 months of age, were obtained from local commercial sources. The fish were maintained in 15-gallon aquaria filled with aerated, conditioned water at 28°C and fed commercial flake food (Tetra) twice daily; each morning and afternoon. This study was carried out in accordance with the Guide for the Care and Use of Laboratory Animals of the National Research Council (USA). All protocols on animal care and experimental procedures were approved by the Institutional Animal Care and Use Committee (project number 16-04-01). A sample size of 3–5 was used for every time point.

### Repetitive Damage to the Olfactory Organ

The method for deafferentation involved the insertion of a wax plug into the rosette, as previously reported (Scheib et al., 2019). Zebrafish (*n* = 30) were anesthetized with 0.03% MS222 (3-amino benzoic acid ethyl ester, Sigma) until unresponsive to a tail pinch. Fish were placed on a chilled putty dish and covered with a chilled paper towel to support them and keep them anesthetized during the procedure. A small ball of medical-grade paraffin orthodontic wax mixed with a trace amount of Methylene Blue powder (for visualization) was inserted into the right naris; the left naris remained unplugged for use as an internal control for comparison. The plugs often fell out as the fish swam (averaged approximately 6 h), so plugs were checked every 12 h and reinserted if lost over the course of 4 h, 12 h, 1 day and 7 days survival times. To examine recovery potential, some fish had plugs removed after 7 days of repeated insertions and were left for 7 days with no plug before euthanasia. Untreated control animals were anesthetized prior to euthanasia on day 0 and were not subjected to wax plug insertions.

### Tissue Processing

After survival times up to 7 days, untreated control fish and treated fish (a minimum of three fish per group for each survival time were used) were over anesthetized with 0.03% MS222 and perfused transcardially with PBS before immersion in 4% paraformaldehyde for 24 h at 4°C. Either dissected brains or decalcified whole heads were rinsed in PBS and mounted in a gelatin and sucrose mixture that was fixed in 4% paraformaldehyde for 24 h at 4°C. The gelatin block was cryoprotected through a gradient of sucrose solutions up to 30% sucrose. Blocks were then flash-frozen in 2-methyl butane, embedded in OCT (Tissue-Tek), and sectioned on a cryostat (Leica CM1860) at 10 μm for rosette analysis, 30 μm for whole bulb and glomerular analysis, or 50 μm for proliferation analysis. Sectioned brains were mounted on Colorfrost Plus (Fisherbrand) positively charged slides or gelatin-covered neutral slides (CEL & Associates Inc., Los Angeles, CA, USA) and vacuum sealed overnight. Olfactory organ morphologies were observed in tissue stained with typical hematoxylin and eosin protocols.

### Immunohistochemistry

An antibody to keyhole limpet hemocyanin (KLH) was used to label olfactory sensory neuron axons and antibodies to GFAP or GS were used to label astroglia in the olfactory bulb. Mounted sections were rinsed in PBS and immersed in a blocking solution of 3% normal goat serum and 0.4% Triton X-100 in PBS for 1 h at room temperature. Sections were incubated for 2 h at room temperature with anti-GFAP (Dako Z0334 made in rabbit; 1:1,000 in blocking solution) or anti-GS (Millipore Sigma MAB302 made in the mouse; 1:1,000 in blocking solution). Slides were rinsed in PBS and incubated in Alexa Fluor 488 goat anti-rabbit or goat anti-mouse IgG (Invitrogen; 1:200 in blocking solution) for 1 h at room temperature or 24 h at 4°C. Following rinses in PBS, sections were incubated in 3% normal rabbit serum for 1 h at room temperature, rinsed in PBS, then incubated in 30 μg/ml Fab fragments (Jackson ImmunoResearch Laboratories Inc., West Grove, PA, USA) for 1 h at room temperature to block any anti-rabbit binding sites. Slides were then rinsed in PBS and incubated with the second primary antibody anti-KLH (Sigma H0892 made in rabbit; 1:1,000 in blocking solution) overnight at 4°C. Sections were rinsed in PBS and incubated in Alexa Fluor 563 goat anti-rabbit IgG (Invitrogen; 1:200 in blocking solution) for 1 h at room temperature. Some slides were rinsed and incubated in Hoescht dye (1:15,000) for 10 min at room temperature to view all nuclei. Slides were rinsed in PBS and coverslipped using a PVA-DABCO mounting solution.

No primary antibody controls were performed to ensure that the observed labeling was specific to the primary antibodies. Slides were incubated overnight at 4°C in blocking solution, rinsed in PBS, then incubated in AlexaFluor 488 goat anti-rabbit as above for 1 h at room temperature. Following PBS rinses, slides were incubated in AlexaFluor 563 goat anti-mouse as above for 1 h at room temperature. Slides were rinsed in PBS and coverslipped using a PVA-DABCO mounting medium.

### Quantitative Analysis of Astroglial Branching and Hypertrophy

Olfactory bulbs were viewed on a confocal microscope (Nikon Ti Eclipse). Maximum intensity projections of Z stacks consisting of 11 optical slices at 2 μm through 50 μm tissue sections were taken of whole bulbs at low magnification and of a ventral medial glomerulus that was consistently found in all samples at high magnification using Nikon C2 Elements software. The full intensity image was viewed using ImageJ software, with the channels split individually into grayscale, and an estimate of the amount of labeling in treated and untreated tissues was obtained by comparing the optical density (OD) of the grayscale representation of antibody labeling. To obtain a mean gray value of anti-GFAP labeling of whole bulbs, the bulb was traced on the GFAP channel and measured. To account for background intensity, a similarly sized area was traced on an unstained area of the slide. To obtain a mean gray value of anti-GFAP labeling associated with the glomerulus, the glomerulus was traced on the KLH channel, the size and position of the trace were maintained when switched to the GFAP channel, and a mean gray value was taken within the trace. Background values for glomeruli were obtained on the GFAP channel by drawing a circle in an area without distinct labeling and with KLH labeling in the glomerulus. These data were converted to an OD using the following formula: OD = −log (intensity of the background/intensity of area of interest). Then, the ODs of a single Z stack projection from each bulb was compared using a percent difference formula: % Difference = (OD of experimental bulb − OD of internal control)/OD of internal control of the same fish. Both the raw OD data and percent difference data were compared using ANOVA with Tukey’s test for multiple comparisons or repeated-measures ANOVA with Bonferroni *post hoc* test on GraphPad software. *P* values less than 0.05 were considered significant.

## Results

### Astroglial Structures in the Olfactory Bulb of Adult Zebrafish

**Figure 1:**
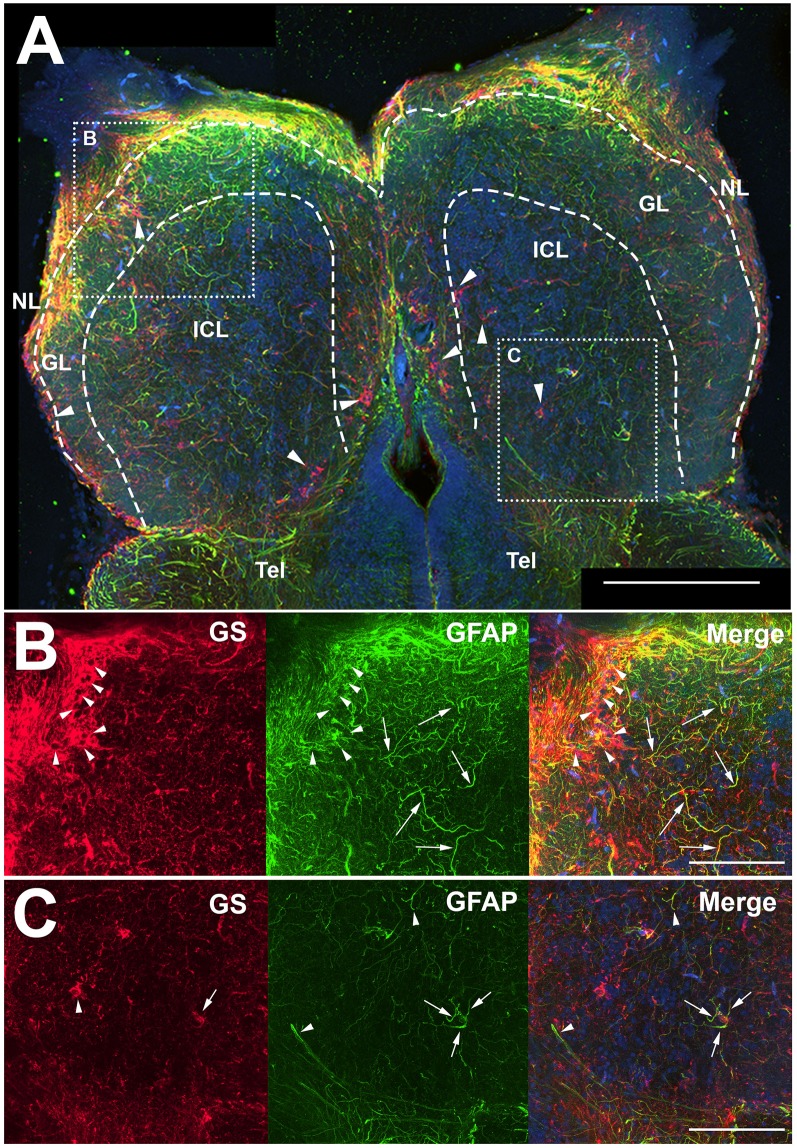
Z-stack images of anti-glial fibrillary acidic protein (GFAP) and anti-glutamine synthetase (GS) immunoreactivity in the olfactory bulb of uninjured adult zebrafish. **(A)** There was a high degree of labeling for both antibodies in the nerve layer (NL) and glomerular layer (GL) of the olfactory bulb that varied in co-labeling. Anti-GS (red) labeling did not reveal clear structures with the exception of ring-like labeling seen in all layers of the bulb (arrowheads). There were numerous, distinct anti-GFAP (green) labeled processes in the GL and internal cell layer (ICL) that varied in size and length but had no obvious organization. Tel, Telencephalon. **(B)** Higher magnification of the rostral bulb region shown in **(A)** confirmed that ring-like structures (arrowheads) were labeled mostly with anti-GS and distinct processes (arrows) were labeled predominantly with anti-GFAP. **(C)** Higher magnification of the caudal bulb region revealed less labeling with both antibodies in this region, but anti-GS positive ring-like structures and anti-GFAP positive processes were apparent. Scale bar = 100 μm **(A)** or 20 μm **(B,C)**.

Antibodies against GFAP and GS were used to identify astroglial structures in the olfactory bulb (Figure 1). Both antibodies labeled structures throughout the uninjured, control olfactory bulb, and the nerve layer (NL) appeared to have a high degree of labeling for both proteins (Figure 1A). The glomerular layer (GL) had many, distinct anti-GFAP labeled processes that varied in co-labeling with anti-GS, and the internal cell layer (ICL) consisted primarily of diffuse anti-GFAP labeled processes. The anti-GFAP profiles did not appear to have any obvious organization but did appear to be denser in the GL than in the ICL. Anti-GS labeling revealed ring-like structures in all layers of the olfactory bulb that, upon higher magnification analysis, consisted of a Hoechst dye-labeled center (Figure 1B). Higher magnification also confirmed our observation that anti-GFAP clearly labeled processes that varied in co-labeling with anti-GS. Antibodies against GFAP did not reveal evidence of a classical, stellate astrocyte morphology as seen in mammals; instead we found only processes that lacked clear organization (Figure 1C). However, some anti-GFAP profiles appeared to terminate on capillaries, a feature that would be expected of astrocytes.

**Figure 2:**
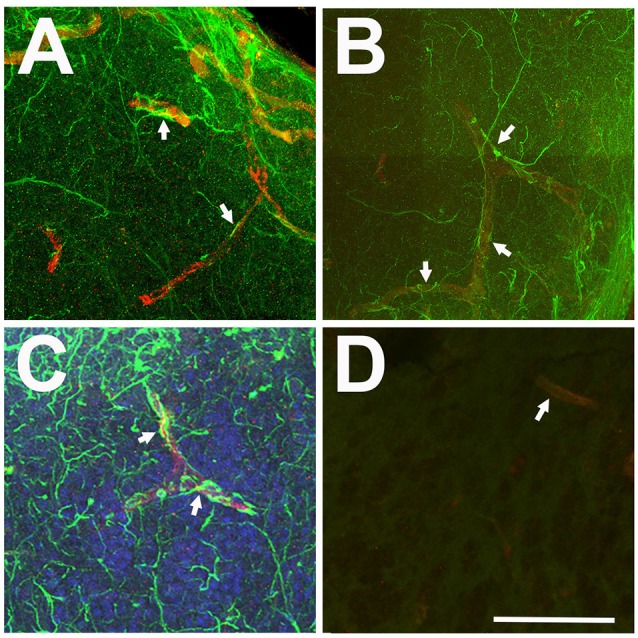
Relationship between anti-GFAP labeling and capillaries viewed in Z-stack images. **(A–C)** Anti-GFAP processes (green) often appeared to terminate onto capillaries (arrows), identified by their tubular appearance and red auto-fluorescence. **(D)** Antibody controls where the primary antibody was eliminated show low levels of green background staining and red auto-fluorescent capillaries (arrow). Scale bar = 20 μm for all.

Only antibodies against GFAP clearly labeled cellular processes. Some of these processes appeared to terminate in endfeet on capillaries (Figure 2). This may indicate an astrocyte-specific physiological role in the regulation of the blood-brain barrier and/or neurovascular coupling (Newman and Volterra, 2004; MacVicar and Newman, 2015; Molofsky and Deneen, 2015; Liu et al., 2018). Since our study focused on morphological changes of astroglial branches, we determined that anti-GFAP antibodies were a superior marker over anti-GS for astroglia and astrogliosis in the adult zebrafish olfactory bulb.

### Repetitive Peripheral Damage Alters Anti-GFAP Labeling

**Figure 3:**
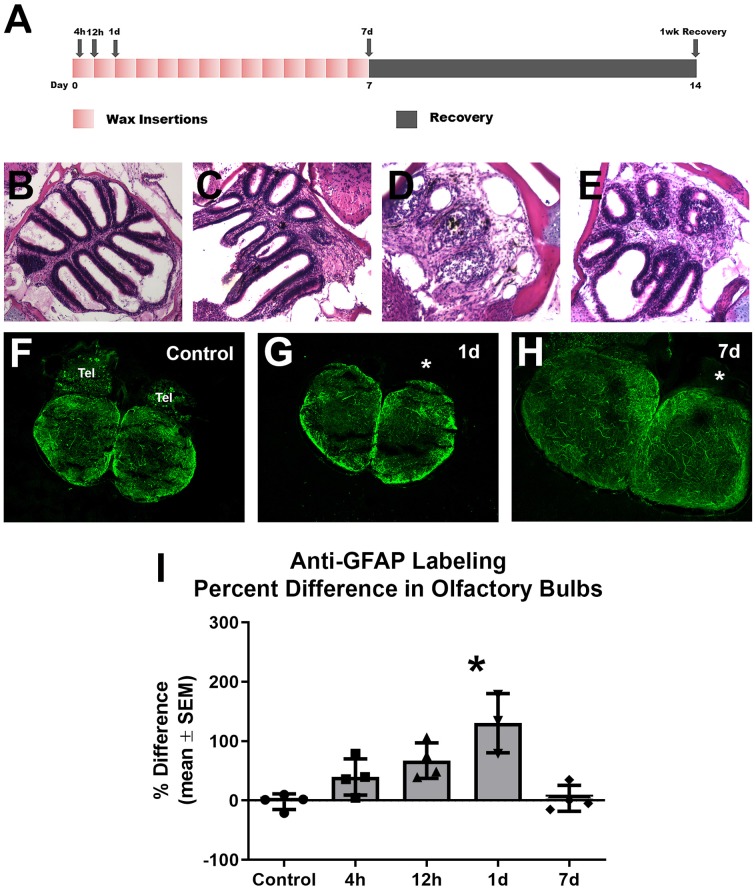
Gross analysis with Z-stack images of anti-GFAP labeling in the olfactory bulb following repetitive peripheral damage. **(A)** Illustration of the treatment paradigm shows that fish receiving repeated wax plug insertions were analyzed at various survival times during wax treatment and after a recovery period. Images of olfactory organs show typical rosette morphology in control fish **(B)**, significant damage at 1 day **(C)** and 7 days **(D)** of wax plug insertions, and return to control morphology with 1 week of recovery **(E)** after removal of the plug. **(F)** Untreated control fish had typical anti-GFAP labeling in all layers of both olfactory bulbs. Tel = Telencephalon, *n* = 4. **(G)** After 1 day of repetitive peripheral damage to the olfactory organ, the affected bulb (asterisk) appeared to have slightly more anti-GFAP labeling than the internal control bulb, *n* = 3. **(H)** By 7 days, there was no noticeable difference in labeling between bulbs, and anti-GFAP labeling in the affected bulb (asterisk) appeared at control levels, *n* = 4. Scale bar = 100 μm for all.**(I)** A comparison of anti-GFAP labeling between bulbs showed a significant difference at 1 day of repetitive peripheral damage when compared to controls but no difference at 7 days of damage. *p < 0.05.

To investigate whether astroglia responds to repetitive peripheral damage, zebrafish were subjected to wax plug insertions into the nasal cavity every 12 h for 7 days to destroy the olfactory organ, and their olfactory bulbs were labeled with anti-GFAP to identify astroglial processes (Figure 3A). The olfactory organ was altered dramatically with wax plug insertions, as shown previously (Scheib et al., 2019). Control rosettes displayed the typical pattern of sensory epithelium lining lamellae (Figure 3B), while 1 day (Figure 3C) and 7 days (Figure 3D) of wax plug insertions caused a progressive deterioration of the organ. With 1 week of recovery after cessation of wax plug insertions (Figure 3E), the olfactory organ returned to control morphology. The pattern of anti-GFAP labeling was similar in both olfactory bulbs in untreated control fish (Figure 3F). While there was no obvious increase in anti-GFAP labeling after 1 day of repetitive peripheral damage, there seemed to be some alteration in the labeling of processes in the GL of the affected bulb (Figure 3G). After 7 days of repetitive peripheral damage, affected olfactory bulbs appeared to have anti-GFAP labeling similar to control bulbs (Figure 3H). To estimate the amount of antibody labeling and determine if there were changes in labeling patterns, OD measurements were made of anti-GFAP labeling within the olfactory bulb. There was no significant difference between left olfactory bulbs of untreated and treated fish throughout the time course (*p* = 0.1379); therefore, these served as internal controls. Labeling was significantly higher in the affected bulb (0.2152 ± 0.0578) compared to the internal control bulb (0.0931 ± 0.0090) at 1 day, and the percent difference in labeling between the bulbs was significantly different (Figure 3I). At 7 days, labeling had returned to control levels and was not different from controls. Thus, the affected bulb had significantly more anti-GFAP labeling at 1 day, yet labeling returned to control levels by 7 days.

**Figure 4:**
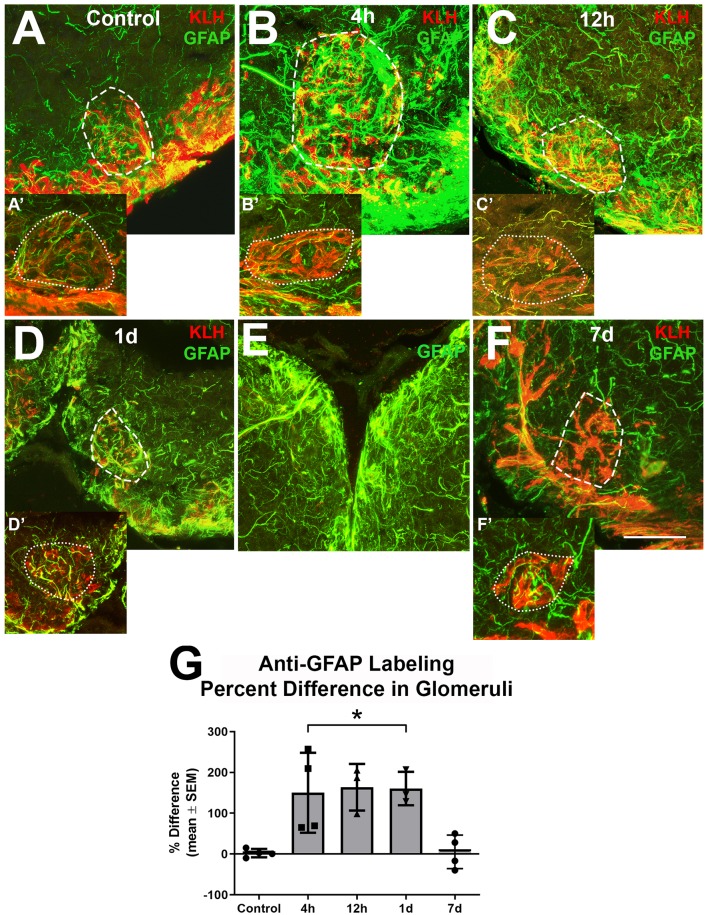
Higher magnification analysis of Z-stacks of anti-GFAP (green) and anti-keyhole limpet hemocyanin (KLH; red) labeling during repetitive damage to the olfactory organ. **(A)** Anti-KLH allowed identification of a ventral medial glomerulus (dashed outline). Anti-GFAP labeled processes within the glomerulus in the right olfactory bulb (dashed outline) varied in size and length and had no clear organization in untreated control bulbs. **(A’)** A glomerulus (dotted outline) in the same region of the left olfactory bulb of the same fish displayed similar morphology of anti-GFAP labeled processes, *n* = 4. **(B)** After 4 h of damage to the olfactory organ, anti-GFAP labeled processes appeared to be more numerous and thicker within the glomerulus (dashed outline) of the affected bulb when compared to the comparable glomerulus (dotted outline) of the internal control bulb **(B’)**, *n* = 4. **(C)** By 12 h after damage to the periphery, there were numerous anti-GFAP labeled processes associated with the glomerulus (dashed outline) in the affected bulb that varied in length and thickness, while the glomerulus (dotted outline) in the internal control bulb **(C’)** retained typical labeling, *n* = 3. **(D)** At 1 day after repeated damage to the olfactory organ, affected glomeruli (dashed outline) still appeared to have thicker and more numerous anti-GFAP labeled processes when compared to glomeruli (dotted outline) in the internal control bulb **(D’)**, *n* = 3. **(E)** Side by side comparison of both bulbs at 1 day showed that the left, internal control bulb retained control levels of anti-GFAP labeling, while the right, treated bulb showed more and thicker labeled processes. **(F)** By 7 days after repetitive peripheral damage, anti-GFAP labeled processes in glomeruli (dashed outline) in the affected bulb appeared to have similar morphology to anti-GFAP labeled processes in glomeruli (dotted outline) of the internal control bulb **(F’)**, *n* = 4. Scale bar = 20 μm for all. **(G)** The percent difference of anti-GFAP optical density (OD) measurements between treated and untreated glomeruli was significantly different at 4 h, 12 h, and 1 day of repetitive peripheral damage but was no longer significantly different after 7 days of repeated damage. *p < 0.05.

Since it appeared that the majority of the alterations in astroglial process density was in the GL, and glomeruli are areas with a high density of synapses where astroglia likely have influence, higher magnification analysis of astroglial processes within glomeruli was done to further explore our observations (Figure 4). Glomeruli were identified by their roughly spherical structure when labeled with antibodies against KLH and a ventral medial glomerulus was chosen for detailed analysis because it is easily identified by its stereotypical shape and location. There were several thin anti-GFAP-positive profiles within glomeruli that varied in size and length in untreated control bulbs (Figures 4A,A′). Four hours after the first wax plug insertion into the nasal cavity there was a distinct increase in the amount and overall size of astroglial branches within the affected glomeruli when compared to the untreated and internal control bulbs (Figures 4B,B′). This same effect was apparent at 12 h (Figures 4C,C′) and 1 day (Figures 4D,D′). While the right, treated bulb showed alterations in GFAP expression, the left, internal control bulb retained control levels of anti-GFAP labeling (Figure 4E). However, astroglial processes appeared to return to typical morphology within glomeruli by 7 days (Figures 4F,F′) of repetitive peripheral injury.

To quantify this observation, OD measurements of anti-GFAP labeling within a specific glomerulus in the ventral medial cluster were compared. There was no significant difference between glomeruli in the left bulbs in untreated and treated fish during the time course (*p* = 0.0810), so these served as internal controls. The mean ODs in glomeruli of affected bulbs at 4 h (0.2010 ± 0.0557), 12 h (0.2010 ± 0.0870), and 1 day (0.1761 ± 0.1305) were significantly higher when compared to the internal control bulbs (4 h = 0.0843 ± 0.0195, 12 h = 0.0735 ± 0.0201, 1 day = 0.0703 ± 0.0549). The percent difference between right and left glomeruli was significantly different at these time points when compared to controls (Figure 4G). The percent difference in labeling at 7 days was not different from controls. Thus, the affected glomeruli had more anti-GFAP labeling during 4 h, 12 h, and 1 day of repetitive peripheral damage, yet returned to control levels by 7 days.

### Lack of Glial Scar

**Figure 5:**
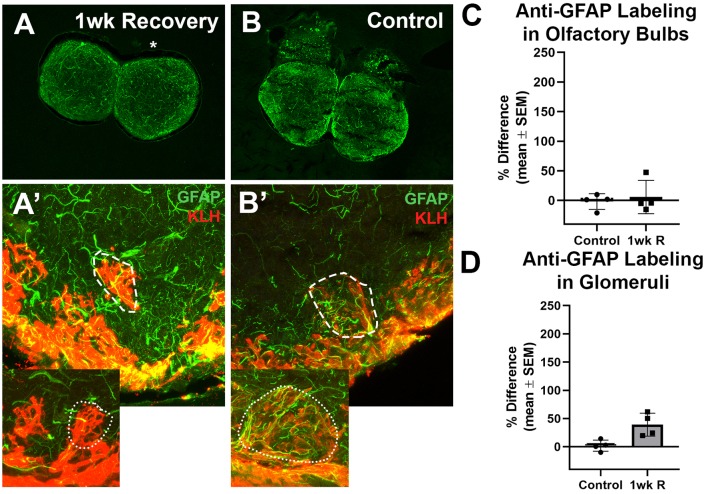
The recovery of the olfactory bulb does not involve evidence of a glial scar. **(A)** After the olfactory system was allowed 1 week to recover from 7 days of repetitive peripheral damage, there was no apparent difference in the overall amount of anti-GFAP (green) and anti-KLH (red) labeling in the affected bulb (asterisk) when compared to the internal control bulb (*n* = 4). There was also no noticeable difference in the overall amount of anti-GFAP labeling compared to untreated control fish **(B)**. **(A’)** Higher magnification Z-stack images revealed that anti-GFAP labeling (green) within anti-KLH (red) labeled glomeruli (dotted line) in the affected bulb after 1 week of recovery also appeared to be similar to that of glomeruli in the internal control bulb (dotted outline in inset) and in untreated control bulbs **(B’)** on the right (dashed outline) and left (dotted outline in inset) sides. There was no significant difference in the percent difference of OD of anti-GFAP labeling in the olfactory bulb **(C)** or ventral medial glomeruli **(D)** of zebrafish that were allowed 1 week of recovery from 7 days of repetitive peripheral damage.

Lastly, to determine if there was any evidence of glial scar formation, zebrafish were allowed to recover for 1 week from 7 days of repetitive damage to the olfactory organ, and their olfactory bulbs were labeled with antibodies to GFAP and KLH (Figure 5). It was previously reported that the removal of wax plugs after 7 days of insertions allows recovery of the olfactory organ and reinnervation of the olfactory bulb within 1 week (Scheib et al., 2019). Anti-GFAP labeling appeared to be similar to untreated controls after 1 week of recovery in whole bulbs and in glomeruli (Figures 5A,B′).

Anti-GFAP labeling in the whole bulb and in ventral medial glomeruli was compared. Following the significant increase in labeling at 1 day of repeated damage reported above, there was no significant difference between the mean OD values of left bulbs of control fish and 1 week recovery fish (*p* = 0.7735) nor right bulb OD means (*p* = 0.8793). There was also no significant difference in the percent difference between bulbs at 1 week of recovery compared to controls (Figure 5C). No significant difference was found in the mean ODs between left glomeruli (*p* = 0.9352) nor right glomeruli (*p* = 0.2553) between controls and 1 week recovered fish (Figure 5D).

## Discussion

### Astroglia in the Olfactory Bulb of Adult Zebrafish

Zebrafish lack evidence of classical stellate astrocytes, but they have glia that expresses the morphology and immunoreactivity of radial glia in most areas of the brain (Grupp et al., 2010). This observation, along with the fact that radial glia can directly differentiate into astrocytes (Malatesta et al., 2008), has led to the inference that radial glia performs the tasks of mammalian astrocytes in the zebrafish brain. However, since the identification and classification of zebrafish glia are incomplete, conflicts in the definitions for these cells have arisen. Typical radial glia does not perform astrocyte tasks, by definition, in mammalian systems, where glial cell types are much better understood. Thus, we classify “astroglia” in this report as the cells that are the most like mammalian astrocytes in the adult zebrafish olfactory bulb, identified by their morphology, immunohistochemical reactivity, and inferred physiological roles.

Anti-GFAP antibodies are commonly used to identify astrocytes and glial scars in adult mammals (Sofroniew, 2009, 2015). Here, we show the immunohistochemical profile of anti-GFAP labeling in the olfactory bulb of adult zebrafish, which lack clearly identifiable cell bodies and organization of glial branches, as previously reported (Byrd and Brunjes, 1995). This morphology is distinct from the organization of anti-GFAP labeling in the zebrafish telencephalon, which shows the morphology of radial glia, and appears to be unique to the olfactory bulb. Another consideration is that olfactory ensheathing cells, a unique glial cell type found in the olfactory nerve, also express GFAP (Barber and Dahl, 1987) and are expected in the olfactory bulb especially following damage (Barnett and Riddell, 2004; Nazareth et al., 2015). In an attempt to elucidate further astroglial morphologies in the olfactory bulb, antibodies against GS, a cytosolic enzyme in astrocytes (Norenberg and Martinez-Hernandez, 1979), were also used. We hypothesized that GS may be expressed specifically in astroglia in the zebrafish olfactory system and not in radial glia or olfactory ensheathing cells. Therefore, anti-GS antibodies were selected in an attempt to eliminate the labeling of anti-GFAP-immunoreactive radial glia and olfactory ensheathing cells from astroglia.

Anti-GS labeling revealed many ring-like structures seen in all layers of the olfactory bulb that are likely cell bodies since they were co-labeled with Hoechst dye at their centers. Since these profiles had some co-labeling with anti-GFAP, they are possibly astroglial cell bodies. However, there was inconclusive evidence to determine that these were, in fact, astroglial cell bodies and, since this study focused on astrogliosis where observations on the size and number of branches were needed, we selected anti-GFAP antibodies as our primary marker for astroglial processes and astrogliosis. Furthermore, since anti-GFAP labeled processes were prevalent in glomeruli, areas of a high density of synapses between olfactory sensory neuron axons and olfactory bulb neurons, and appeared to terminate on capillaries, we concluded that anti-GFAP antibodies were a more reliable marker for astroglia, because the location of immunoreactive processes suggests physiological roles, such as communication at synapses and with capillaries. Therefore, the remainder of this study focused on astroglial branches identified using anti-GFAP antibodies and proliferating glial cells identified using anti-GFAP and anti-PCNA antibodies.

### Astrogliosis in the Presence of Repetitive Peripheral Damage

Adult zebrafish were subjected to wax plug insertions into the right nasal cavity to crush the olfactory organ every 12 h for 7 days, while the left side served as the internal control. This technique results in a significant reduction in olfactory organ size and structure and reduces afferent innervation of the olfactory bulb (Scheib et al., 2019). Astroglial morphology and the amount of anti-GFAP labeling in the olfactory bulb were investigated. Qualitative and quantitative analyses revealed an increase in the size and number of branches in and around glomeruli during timepoints up to 1 day of repeated damage when compared to the internal control bulb and untreated control fish.

Comparisons of anti-GFAP labeling were made between affected and internal control sides of whole olfactory bulbs and also of individual glomeruli. There was no significant difference in OD among left sides, which served as external and internal controls throughout the time course; therefore, the left side was shown to be unaffected by insult to the right bulb. There was a significant increase in OD of labeling in the affected bulb at 1 day that was likely due to increased amount and size of astroglial branches, which is typical of astrogliosis (Sofroniew and Vinters, 2010). The wax plug caused deterioration of olfactory sensory neurons (Scheib et al., 2019) and, since their axons project to glomeruli in the bulb where astroglial processes were seen, astroglia likely detects this insult and respond with astrogliosis. Since the literature on zebrafish astrocytes, astroglia, and olfactory ensheathing cells is severely lacking, the identity of these anti-GFAP-immunoreactive proliferating profiles remains unclear.

Astrogliosis increased to a significant level by 1 day and attenuated morphologically by 7 days of damage. This is not typical of what would be expected of mammalian astrocytes in a chronic injury environment, which would have retained their astrogliosis morphology and formed glial scars (Ojo et al., 2016; Kulbe and Hall, 2017). This is the first study on astrogliosis during repetitive insults to the zebrafish olfactory system, and the apparent attenuation of astrogliosis is a novel finding.

Repetitive peripheral injury was insufficient to cause astroglial scarring in the olfactory bulb of zebrafish. Insults to other areas of the zebrafish nervous system have been reported previously to be insufficient to cause a glial scar formation (Kroehne et al., 2011; Baumgart et al., 2012; Noorimotlagh et al., 2017), suggesting unique differences in injury response between teleosts and mammals. There was no noticeable difference in the amount of anti-GFAP labeling after 1 week of recovery from repetitive peripheral damage between affected and unaffected bulbs, meaning there was no evidence of a glial scar, adding to the evidence of dynamic zebrafish neuroplasticity. Newly proliferated astrocytes typically form glial scars in mammals (Wanner et al., 2013), therefore we next investigated astroglial proliferation using this model.

These data suggest that astrogliosis in the adult zebrafish olfactory system occurs after a repetitive peripheral injury that attenuates and leaves no residual glial scar. Future studies will examine if astroglial proliferation occurs in response to this damage. This novel finding suggests that astrogliosis differs in this zebrafish repetitive damage model compared to mammals (Kane et al., 2012; Petraglia et al., 2014; Kulbe and Hall, 2017), where repetitive insults results to the brain can cause chronic astrogliosis and the development of protein tangles and plaques. Since astrocytes are such dynamic cells in the CNS, perhaps chronic astrogliosis causes the secondary damage necessary to cause the development of these disease states. If so, understanding astroglia and astrogliosis in zebrafish might lead to novel medical treatments for humans sufferers of these diseases.

## Data Availability Statement

The raw data supporting the conclusions of this article will be made available by the authors, without undue reservations, to any qualified researcher.

## Ethics Statement

The animal study was reviewed and approved by Western Michigan University Institutional Animal Care and Use Committee.

## Author Contributions

JS and CB-J conceived and designed the study, analyzed and interpreted the data and contributed to manuscript revision, read and approved the submitted version, and agree to be accountable for the content of the work. JS collected data and wrote the first draft of the manuscript. CB-J obtained funding for the work.

## Conflict of Interest

The authors declare that the research was conducted in the absence of any commercial or financial relationships that could be construed as a potential conflict of interest.
